# Meat food fraud risk in Chinese markets 2012–2021

**DOI:** 10.1038/s41538-023-00189-z

**Published:** 2023-04-03

**Authors:** Xiaoman Li, Mingwu Zang, Dan Li, Kaihua Zhang, Zheqi Zhang, Shouwei Wang

**Affiliations:** Beijing Key Laboratory of Meat Processing Technology, China Meat Research Center, Beijing Academy of Food Sciences, 100068 Beijing, China

**Keywords:** Social sciences, Science, technology and society, Business and industry, Industry

## Abstract

Food fraud is a major concern worldwide, and the majority of cases include meat adulteration or fraud. Many incidences of food fraud have been identified for meat products both in China and abroad over the last decade. We created a meat food fraud risk database compiled from 1987 pieces of information recorded by official circular information and media reports in China from 2012 to 2021. The data covered livestock, poultry, by-products, and various processed meat products. We conducted a summary analysis of meat food fraud incidents by researching fraud types, regional distribution, adulterants and categories involved, categories and sub-categories of foods, risk links and locations, etc. The findings can be used not only to analyze meat food safety situations and study the burden of food fraud but also help to promote the efficiency of detection and rapid screening, along with improving prevention and regulation of adulteration in the meat supply chain markets.

## Introduction

Economic motivation adulteration (EMA) refers to an action when someone uses food to deceive illegally for economic gain, which was early academically by Spink and Moyer. EMA belongs to a subcategory of “food fraud”^1^. Moreover, the working definition of food fraud (FF) given by the US Food and Drug Administration is “the fraudulent, intentional substitution or addition of a substance to the product, to increase the apparent value of the product or reduce the cost of its production”^2^. The term is not the same as the food fraud definition used in the EU, which refers to “any suspected intentional action by businesses or individuals to deceive purchasers and gain undue advantage, in violation of the rules referred to in Article 1(2) of Regulation (EU) 2017/625 (the agri-food chain legislation)”. And the fraud classification was divided into substitution, dilution, concealment, counterfeit, grey market, forgery, unapproved enhancement, and mislabeling, which covers many more aspects^3^.

Records of food fraud events appeared very early, such as adding alum to bread, gypsum, and starch to milk, etc.^4,5^. With the development of modern industrialization and the expansion of the market scale, the increasing food types have brought more complex changes in products. Events dating back to more than 10 years ago, such as the widely known lawbreakers intentionally mixing melamine into wheat gluten used in pet food processing and infant formula milk powder^6^, show that food adulteration and fraud are increasingly becoming an issue of global concern. According to records, meat adulteration or fraud is one of the highest proportions of all food-related cases^7,8^. The horsemeat scandal that broke out in 2013 further exposed the vulnerability of the global meat supply chain, and raw meat faced a greater risk of adulteration^9–11^. A similar incident occurred in 2017; Brazil’s weak meat scandal has seen plenty of substances and means, including the substitution of animal-derived ingredients, excessive food additives, and water injection^12–14^. The many food safety incidents in China, such as clenbuterol-tainted meat products in 2011, have threatened consumers’ health and trust in the food supply.

The constant trend is that Chinese consumers are increasing their consumption of animal-based foods due to good nutritional and sensory properties, especially preferring more beef and mutton over the past decade^15^. From 2011 to 2020, per capita, household consumption of livestock and poultry meat in China increased by 22.8%^16^. The meat in the market mainly comes from domestic production and imported meat, both of which are growing in quantity. In particular, the growth rate of imported beef and mutton is very prominent (128.9% of imported meat, 235.2% of imported beef, and 64.9% of imported mutton in the recent 5 years)^17^. From the perspective of domestic meat products, since ancient times, China’s unique processing method of meat products has derived 5 categories and more than 500 sub-categories of traditional processed meat products suitable for the local taste, including sauce-braised products, cured meat products, dried meat products, smoked barbecue products, and fermented products^18^. Moreover, the annual consumption of sauce-braised meat products can reach 50 million tons. However, in fact, since the 1980s, the slaughter industry standardization system has only just developed^19^. The unbalanced economic development between production areas, as well as differences in the level of safety control, have led to an increase in supply chain vulnerabilities. Thus there are still many food fraud events such as unapproved enhancements, adulteration, and counterfeiting continuing to occur during the slaughter, circulation, or other links, which poses challenges to the protection of consumers and management of food safety^20,21^.

Understanding and documenting food fraud incidents will help to share the relevant information, target resources, and prevent the occurrence of such affairs^22^. In the wake of the horse meat adulteration incident, the European Commission has established the EU Food Fraud Network, where government agencies could share information and intelligence on incidents, and has set up a new dedicated category of food fraud to the RASFF system^23,24^. Simultaneously, many agencies and organizations have established food fraud incident databases, such as the Decernis’ Food Fraud Database (the formerly United States Pharmacopoeia Food Fraud Database)^25^, HorizonScan Database from the UK Food and Environment Research Agency^26^, the Food Protection and Defense Institute’s Food Adulteration Incidents Registry^27^, and FoodSHIELD’s Food Adulteration Incident Registry (FAIR) Database^28^, etc. Almost all of them have compilated historical and current incident data involving FF on a global or regional scale. Scholars have conducted an in-depth analysis of the data in the above databases^13,14,29^ or provided early warning of FF behavior through risk management methods and data models constructed^21,30^. Moreover, others analyzed the characteristics and rules of relevant incidents based on the statistical data obtained, For example, they analyzed food adulteration cases in China based on media information reports^8^, reviewed the occurrence of FF events based on academic journal articles^29,31^, and analyzed food fraud problems based on the data published by the government in the notice of supervision and sampling inspection^32,33^.

Studies on meat food fraud in target markets are also critical to the prevention and mitigation of fraud, as well as the detection and certification of adulterated ingredients^34,35^. There is a rare systematic analysis and research of meat food fraud. At the same time, due to the limited inclusion of local meat food FF events in international databases, our previous research on food fraud only collected related official supervision inspection data from China^32,33^. The study believes that it is not enough only collect information from supervision and sampling data or a single source of media information, and it is necessary to combine the two. Moreover, there are many imported meat foods on the market. Therefore, based on local media reports and official circulars (including data on imported meat products), we have attempted to describe the types of fraud, regional distribution, adulterants and categories involved, categories and sub-categories of foods, risk links and locations identified in these incidents as follows.

## Results

### Regional distribution of FF cases in meat products

**Fig. 1:**
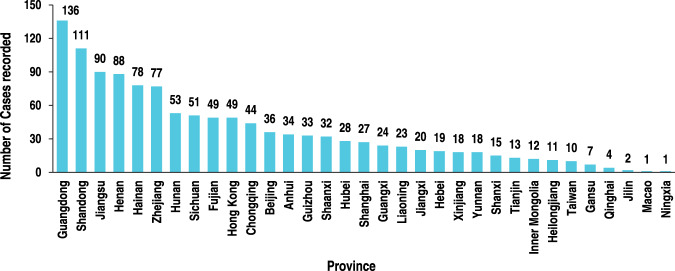
Province distribution of meat FF cases reported in domestic news and supervision & sampling inspection notifications. The columns indicate the total number of incidents in each province checked or reported as substandard based on the adulterants.

Our results showed that the 1214 samples of domestic meat fraud occurred in more than 33 provinces and special administrative regions. The regional distribution is shown in Fig. 1, and the reported cases vary widely among regions. Guangdong (11.2%), Shandong (9.1%), and Jiangsu (7.4%) were the highest, while Gansu (0.6%), Qinghai (0.3%), and Ningxia (0.1%) correspondingly the lowest. This indicates that the occurrence of meat food fraud may be related to the development of the meat industry in this region. According to the China Statistical Yearbook (2020)^16^, Chongqing, Guangdong, and Sichuan are the provinces with the highest household meat consumption, with per capita meat consumption for households of 35.3, 33.6, and 33.6 kg, respectively. Moreover, Shandong, Sichuan, and Henan are the provinces with the highest meat output in China, with annual outputs of 7.3, 6.0, and 5.4 million tons, respectively. Due to the larger scale of meat production and consumption, or higher industrialization level, the proportion of the total sample size of food safety and the corresponding number of news reports would be higher. In contrast, the number of FF reports is relatively low in less developed regions.

**Fig. 2:**
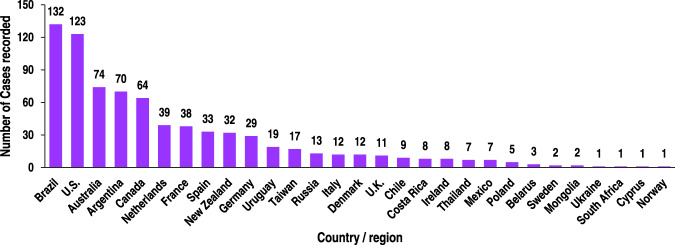
Countries/regions of reported meat FF cases from imported food inspection notifications. The columns indicate the total number of incidents in each country/region checked or reported as substandard based on the adulterants.

Similarly, according to imported meat food FF samples of 773 cases, there were more rejection circulars and news about FF from Brazil (17.1%), the United States (15.9%), and Australia (9.6%) reports (Fig. 2). In 2021, the top five countries in China that imported meat and meat preparations most are Brazil, Spain, the USA, Argentina, and Australia. The import amount reached 51.5, 22.7, 21.7, 16.3, and 15.3 billion yuan, respectively, in 2021^17^. It can be seen that the fraud risk of imported meat is mainly related to the quantity of imported meat.

### Number of FF recording cases per type

We calculated the number listed in Table 1 involving various fraud types and sources of information as described in methods, including imported food products. “Artificial enhancement” accounted for the highest proportion, with 869 cases (43.7%), followed by “illegally imported meat” (40.9%), “replacement” (12.6%), and “counterfeit” (1.0%). Artificial enhancements mostly came from official sampling information (636 of a total of 869 cases) because they were mostly supervised through production, processing, and other links and confirmed by the results of experimental instruments and rapid detection technology. The development of authenticity identification technology promoted qualitative and quantitative analysis of adulterated beef and mutton meat substitution for other animal-derived ingredients, such as the detection of pork and chicken DNA^36^. However, as a technology to identify food fraud or food crime, authenticity identification is not within the scope of daily food safety supervision and sampling in China. Such illegal incidents are mainly found in by media reports information. Furthermore, dilution or other types were more counted by media reports likewise, for their frequent occurrence in the illegal business process that is difficult to find. This is further confirmed by the information on the dopant substances (relevant risk links and locations) in the following text. It can be seen that the media can supplement the fraud report information outside the official inspections and play a key role in patching omissions. For example, among the 813 cases of meat fraud related to illegal imports, 729 were the results of imported food inspection reports released by GAC, and 79 were the cases reported by the media because they may involve some incidents of illegal entry about smuggled meat.

**Table 1:** Statistics on the number of each fraud type in meat foods.

Fraud types	Number of cases involved	Domestic food	Imported food	Media news reports	Percentage (%)
Artificial enhancement	869				43.7
		636	42	191	
Substitution	250				12.6
		15	2	233	
Mislabeling/misbranding	15				0.8
		7		8	
Counterfeiting (IPR)	19				1.0
		2		17	
Dilution	18				0.9
		5		13	
Other	1				0.1
				1	
Certificate fraud	2				0.1
				2	
Illegal imports	813	5	729	79	40.9
Total	1987	670	773	544	100.0

Source: author’s calculation.

### Categories and sub-categories of meat foods involving FF

Knowing which kinds of foods are prone to fraud will help with risk monitoring and applying food safety testing. There are two kinds of meat foods on the market: fresh and frozen livestock, poultry meat, and by-products (unprocessed meat products), as well as processed meat products. According to the classification regular pattern of food categories in the public data of supervision and sampling inspections and our previous literature^32,33^, for fresh and frozen livestock, poultry, and by-products, we divided the product categories into livestock, poultry, poultry by-products, and livestock by-products. For processed meat products, our classifications are cooked meat products, prepared meat products, and catering foods. The categories and sub-categories, as we have shown in Table 2, are parallel without overlapping by listing the number of cases involved. These figures show that among the livestock and poultry meat and their by-products, beef with high sales volume (24.3%), pork (15.9%), other livestock by-products (9.3%), mutton (7.7%) and chicken (7.1%) faced more fraud problems; In addition, the sauce-braised meat products (8.1%), and cured meat products (3.4%) loved for their taste in the cooked meat products also had similar problems. Homemade sauce-braised meat products (5.0%) also have more fraud activities, usually produced by catering operators.

**Table 2:** Categories and sub-categories of meat products involving FF.

Food kinds/categories/sub-categories	Number of cases involved	Percentage (%)
Fresh and frozen livestock, poultry meat, and the by-products	1506	75.8
Livestock	967	48.7
Beef	482	24.3
Pork	315	15.9
Mutton	152	7.7
Meat from other livestock	18	0.9
Poultry	168	8.5
Chicken	140	7.1
Duck	18	0.9
Meat from other poultry	10	0.5
Livestock by-products	235	11.8
Pork liver	27	1.4
Pig kidney	16	0.8
Sheep kidney	6	0.3
Beef liver	2	0.1
Other livestock by-products	184	9.3
Poultry by-products	136	6.8
Chicken liver	5	0.3
Other poultry by-products	124	6.2
Goose liver	2	0.1
Duck liver	5	0.3
Processed meat products	481	24.2
Cooked meat products	272	13.7
Sauce-braised meat products	161	8.1
Dried meat products	44	2.2
Smoked boiled sausage and ham products	44	2.2
Smoked barbecue meat products	23	1.2
Prepared meat products	81	4.1
Cured meat products	67	3.4
Quick-frozen prepared meat products	14	0.7
Catering food	128	6.4
Sauce-braised meat products (homemade)	100	5.0
Other cooked meat (homemade)	22	1.1
Meat enema (homemade)	5	0.3
Aspic, skin jelly (homemade)	1	0.1
Total	1987	100.0

Source: author’s calculation.

### Involving adulterants and categories

Similarly, understanding the risks of adulteration means and adulterants in meat products of different food categories is conducive to improving the transparency of the supply chain and exploring the traceability of related products, and providing a reference for the prevention and control of FF problems^37^. In Table 3, we summarized the fraud types and categories of adulterants, listed in descending order by frequency of reporting. Disqualification of microorganisms was excluded from the adulterants because such behavior was not FF^1,38,39^. Excessive use of food additives and veterinary drug residues were also excluded according to our previous research^32^. Since some cases have involved specific adulterants and circumstances, we will discuss them in detail later.

**Table 3:** Categories of FF adulterants involved in meat foods.

Fraud types/categories of adulterants	Number of cases involved	Percentage (%)
Artificial enhancement	869	43.7
Forbidden veterinary drugs	422	21.2
Misuse of food additives	297	15.0
Industrial substances	93	4.7
Other non-edible substances	57	2.9
Substitution	250	12.6
Adulterate other animal ingredients	151	7.6
Sick/dead livestock & poultry meat	43	2.2
Expired meat (spoiled meat)	31	1.6
Entrained low-quality meat	7	0.4
The meat of unknown origin	6	0.3
Poisonous meat ingredients	5	0.3
Plant-derived ingredients	4	0.2
Unclean meat	3	0.2
Dilution	18	0.9
Water-injected meat	18	0.9
Counterfeiting (IPR)	19	1.0
Counterfeit brand products	16	0.8
Counterfeit products’ origin	3	0.2
Mislabeling/misbranding	15	0.8
The label does not match a product	7	0.4
Unlabeled food additives detected	6	0.3
Missing Labels	2	0.1
Illegal imports	813	40.9
Imported products with no official inspection and quarantine certificate, approval or origin certificate	111	5.6
Not obtained inspection and quarantine access	232	11.7
The certificate does not conform to the goods	470	23.7
Other	1	0.1
Foreign substances	1	0.1
Total	1987	100.0

Source: author’s calculation.

#### Involving prohibited drugs and excessive drug residues

Breeding is an indispensable supporting industry for China’s agriculture. In 2020, the number of large livestock at the end of the year in China reached 102.7 million, and the meat output achieved 77.5 million tons. However, the unqualified products reflected the problems of the breeding source. According to our data (in Supplementary Table 1), many enterprises have feed abuse and illegal use of prohibited drugs in the process of livestock and poultry breeding: (1) The doping prohibited drugs (47.4%) were the most frequently detected, followed by antibiotics (38.9%) and banned pesticides (11.1%); (2) In particular, the problem of clenbuterol (27.0%), ractopamine (17.5%), chloramphenicol (16.4%), sodium pentachloro phenate (11.1%) and ofloxacin (8.8%) in meat are prominent. The basis was mainly from *the List of Drugs and Other Compounds Prohibited from Use in Food Animals* published by the Ministry of Agriculture and Rural Affairs Announcement No. 250.

*β*-agonist drugs, clenbuterol, ractopamine, and salbutamol, called “lean meat powder”, belong to the category of doping and are the main problem of animal-source food fraud in China. They helped to improve the meat-to-feed ratio of products and lean meat rate so as to obtain more economic benefits. There is more ractopamine in imported meat and by-products because it is still prohibited in China. These components accumulate most in animal viscera, such as pig liver, sheep kidney, etc. Eating meat and viscera containing those drugs will cause harm to the human body, mainly manifested as accelerated heart rate, metabolic disorders, decreased blood potassium, muscle tremors, headache, nausea, vomiting, and other symptoms^40,41^. In the 1990s, clenbuterol was widely used in pig breeding. Therefore, in 1997, China issued a document to prohibit the use of clenbuterol in feed and animal husbandry and constantly strengthened the control and criminal sanctions against such illegal drug use^42^. However, from 2017 to 2021, there were still many high-frequency clenbuterol detections in meat products through our data results. According to the obtained media news, in 2011, China Central Television (CCTV) exposed that a large enterprise in Henan had purchased “lean meat essence” pork from farmers for many years^43^. By 2021, CCTV also reported the problem of some dealers in Hebei Province selling mutton mixed with clenbuterol^44^. It indicates a trend of intense use of drugs from pork to beef and mutton of the drugs. Detection in the meat of clenbuterol from different species of animals is related to the high economic benefits of the products and low illegal costs. Farmers reflected that once they sold mutton with clenbuterol, they could increase their profits by tens of millions of yuan through large sales.

Further, the illegal addition of other prohibited antibiotics was common in pork and chicken (especially black-bone chicken). Antibiotic chloramphenicol has been detected multiple times in chicken, pork, and cured meat products, but long-term and large-scale consumption of it may cause intestinal flora imbalance, leading to digestive disorders^45^. Ofloxacin and furazolidone metabolites are also overused in chicken and pork farming. Prohibited pesticides sodium pentachloro phenoxide was detected more in chicken and pig liver. In addition, the illegal use of banned antiviral or anti-infective human drugs such as amantadine and ribavirin in aquaculture production was also a common fraudulent substance in animal husbandry. *The Food Safety Law*, *the Animal Husbandry Law*, and *the Criminal Law* clearly stipulate that illegal business operations in violation of national regulations may even be sentenced to more than five years of imprisonment. However, frequent incidents show that despite strict legislation, there is still a lack of corresponding supervision and control in the supply chain, especially in the source link of aquaculture.

#### Involving food additives

Illegal use of additives was also a particularly common fraud. As in Supplementary Table 2, (1) colorants (36.0%), color protectants (22.2%), preservatives (21.2%), bleaching agents (16.5%), and sweeteners (2.0%) were used most in meat food processing; (2) Nitrite, sulfur dioxide, benzoic acid, sunset yellow, sorbic acid and potassium salt were added and used more in the processing of meat products, accounting for 21.9%, 16.5%, 16.2%, 8.1%, and 4.4% respectively. According to the National Food Safety Standard *GB-2760-2014—Standard for the Use of Food Additives*, this kind of additive should not be used in meat products or dishes (for example, nitrite is not allowed to be added in homemade catering meat products). The standard also stipulates that no food additives, spices, and flavors shall be added to raw meat.

Super-range use food additives were to improve the product’s appearance (colorants such as caramel color, Allura red & Monascus red) or to cover up the quality and characteristics of inferior materials (carmine and compound additives), longer shelf life (preservatives such as benzoic acid, sorbic acid & nitrite) or better taste (flavoring agents such as acesulfame potassium, cyclamate, and sodium saccharin). As a water-soluble azo pigment, the excessive use of carmine in meat products may cause carcinogenic and mutagenic effects^46^. It was shown that nitrite would oxidize low hemoglobin to high hemoglobin after entering the blood and generate nitroso compounds, which can cause cancer or acute poisoning^47^. Additives added to meat products during processing may be due to technical requirements. The scope of allowed use of food additives in various countries is different due to differences in the standards and capabilities of independent assessment of food additives in various countries. However, no matter whether these additives are allowed to be used in food or not and whether they cause health hazards, as long as they are abused or added outside the scope of the standard, they are illegal.

#### Involving non-edible substances

There were also many cases involving using non-edible substances, and the proportion of industrial chemicals reached 62.0% (see Supplementary Table 3). Others accounted for 31.3%. The following substances have been reported to be added: poppy shell (31.3%), hydrogen peroxide (16.7%), industrial rosin (14.7%), industrial sodium nitrite (10.7%), formaldehyde (1.2%), industrial sodium nitrite (0.9%), etc.

Most of these substances are identified as non-edible substances (containing some industrial substances in the list) according to the *List of Non-edible Substances and Food Additives that May Be Illegally Added to Food* (batches 1–6) released by the former Ministry of Health in China, such as poppy shells used in small restaurants to pickle meat, hydrogen peroxide used illegally to bleach sheep’s hoofs, industrial rosin used to remove animal hair, and borax added in processing to improve the taste of homemade meatballs, etc. China has stepped up efforts to investigate and punish illegal and criminal acts of non-food material processing and, in particular, fight against such food fraud. With the intensification of crackdowns, this kind of adulteration has improved. However, there are still many mobile workshops and small restaurants that turn to food fraud and crime for improper financial gain. In the case of the poppy shell, as an example, overeating will cause symptoms such as chills, fatigue, sweating, yellowish face, and thin muscles. In severe cases, it will damage the nervous system, respiratory system, and digestive system.

In terms of industrial substances, details in our data indicated that the use of these substances as adulterants were intended to replace legally permitted additives for cost savings (such as industrial substances hydrogen peroxide, formaldehyde) and illegal processing operations such as hair removal (industrial rosin), to alter the color, appearance, or even bleaching of food (such as using acid orange II, alkaline bright yellow O, Sudan red, Congo red, industrial dyes, industrial sodium nitrite, sodium hydroxide, hydrogen peroxide), also to alter texture or stability (such as the use of borax, industrial gelatin, and industrial magnesium chloride). Moreover, most of the substances are used in the processing and sales of livestock and poultry by-products; for instance, there were cases in which formaldehyde was added into cattle louver, cattle gluten, sheep miscellaneous (sheep viscera), pig blood, and duck blood. The above behaviors seriously violate *the Food Safety Law* and *the Product Quality Law*, which stipulates that adulteration, counterfeiting, and other behaviors shall be strictly prevented in the production and processing of products. According to research, formaldehyde in food can cause gastrointestinal discomfort, damage people’s liver function in severe cases, and has potential carcinogenicity^48^. Other banned substances mentioned above may also have similar adverse health effects.

#### Involving meat from other species

The problem of “adulteration” in animal-derived products, that is, the use of cheap meat or tissues from other species to replace higher-priced meat or by-products, has always been the focus of consumer complaints and a hot spot of social media concern. An analysis of species determination of nearly 1000 meat products shows that nearly 20% of product labels do not exactly match the variety^49^. According to the survey data in 2019, the overall adulteration ratio of meat skewers in the local market is 17.5%, among which the adulteration ratio of mutton skewers is 21% and that of beef skewers is 14%^50^. The behaviors are not in conformity with the provisions of *the Food Safety Law*, *the Agricultural Products Quality and Safety Law*, and other laws. Based on our statistics in Table 4, meats with better nutritional content and high prices in the Chinese market, such as mutton (35.1%), beef (33.1%), and dried meat products (8.6%), are the main target ingredients to be substituted/adulterated.

**Table 4:** Main target meat foods substituted by other animal-derived ingredients.

Target ingredients	Numbers of cases involved	Percentage (%)
Mutton	53	35.1
Beef	50	33.1
Dried meat products	13	8.6
Other poultry by-products	9	6.0
Meat from other livestock	6	4.0
Pork	4	2.7
Sauce-braised meat products	4	2.7
Quick-frozen prepared meat products	3	2.0
Sauce-braised meat products (homemade)	3	2.0
Smoked barbecue meat products	2	1.3
Other livestock by-products	2	1.3
Smoked boiled sausage and ham products	1	0.7
Meat from other poultry	1	0.7
Total	151	100.0

Source: author’s calculation.

The major adulterated animal sources found and reported mainly contain pig (2.4%), duck (2.4%), chicken (0.7%), female pork (0.4%), fox (0.4%), etc. While the minor adulterated animal sources, in addition to other possibly adulterated animal-derived ingredients, mainly include by-products of the target counterfeit ingredients such as blood, offal, etc., as well as meat extracts and edible flavors (Table 5).

**Table 5:** Major and minor FF ingredients of other animal origins in meat foods.

Major adulterated animal ingredients	Minor adulterated animal ingredients	Number of cases involved	Percentage (%)
Pig	Chickens, ducks, mules, other livestock; beef lung, sheep oil, butter, beef offal	56	37.1
Duck	Goose, pig, fox; butter, sheep oil, mutton meal	44	29.1
Chicken	Duck, pig, chicken blood, sheep tail oil, pork essence, beef essence	14	9.3
Sow	Dog; beef fat	8	5.3
Fox	Mink, mouse	8	5.3
Mule	Horse, pig	3	2.0
Horse	Donkey, mule, sow	3	2.0
Cattle	Horse, duck	3	2.0
Buffalo	Cow, pig, mouse	1	0.7
Goose	–	1	0.7
Raccoon	–	1	0.7
Cat	–	1	0.7
Other ingredients of unknown animal origin	–	8	5.3
Total		151	100.0

Source: author’s calculation.

Among them, the meat used for adulteration is mostly pork and duck meat, possibly accompanied by minor ingredients such as sheep tail oil, butter, and starch, which are usually processed into fake “mutton rolls” and “beef rolls” sold to the catering link^51^; a small amount of beef jerky and beef granules are also made from pork jerky and added with beef flavor essence; some businesses use pig blood mixed with formaldehyde to replace duck blood for sale (duck blood is more expensive in the market, and is poultry by-product with certain nutritional value)^52^; some unscrupulous traders will also buy fox, mink, mule and other dead livestock and poultry meat, which has not been inspected and quarantined, added with gelatin or nitrite for “bonding” and “color-changing” to be sold as beef and mutton^53,54^. The above behaviors are all motivated by economic interests, replacing high-priced animal-derived products with other animal-derived ingredients of cheap or even unknown origin and then selling them at a price lower than the average price of normal products in the market^55,56^. These adulterated meats involve food fraud, which may lead to epidemic risk. Sometimes it not only involves economic and food safety issues but also directly affects consumers’ health, even religious beliefs.

#### Involving other low-priced alternative meat and ingredients

We regard the use of expired meat or spoiled meat, meat of unknown origin, and poisonous meat ingredients to replace normal meat food as other low-priced alternative meat and ingredients. As shown in Supplementary Table 4, most of the news about sick and dead livestock and poultry as raw materials was reported by the media. According to statistics, these meats are even processed by unscrupulous merchants into bacon, sausage, offal soup, and other products. Expired (deteriorated meat) materials often had quality problems, such as mold, black color, expired, etc. Under suitable conditions, microorganisms will proliferate. When pathogenic microorganisms and their toxins enter the body, they cause poisoning^57^. The poisonous meat ingredients mainly refer to poisoned dog meat. Other low-priced substitutes involved chicken skin, animal lymph, thyroid, etc. In addition, there are alternative ingredients from plant-derived ingredients, but used as raw meat or to add weight^58^.

#### Involving illegally imported meat

As we described in Table 2, illegally imported meat includes products not obtained inspection and quarantine access, lack of official quarantine and origin certificates, or non-conforming goods. Beef (31.4%), pork (24.0%), and by-products (23.6%) were the main sources of the problem (Table 6), which were generally returned or destroyed.

**Table 6:** Products involving FF in illegally imported meat foods.

Products involved	Number of cases involved	Percentage (%)
Beef	255	31.4
Pork	195	24.0
Livestock by-products	112	13.8
Poultry by-products	80	9.8
Chicken	71	8.7
Mutton	42	5.2
Duck	2	0.2
Meat preparations and dishes	52	6.4
Others	4	0.5
Total	813	100.0

In addition, since China’s meat supply has been in a tight balance for a long time, with the growth of domestic consumption demand, consumers’ excessive trust in foreign food, and the difference in meat prices at home and abroad, illicit meat has also become an important area targeted by illegal producers and operators. News media have also reported a number of cases involving the sale and processing of smuggled meat, mostly involving the smuggling of frozen meat, frozen chicken feet, pig feet, and pig kidneys. These products are susceptible to disease and food safety risks due to the lack of inspection reports as well as the difficulty in meeting hygienic standards for transportation and appliances.

#### Counterfeiting (IPR)

Of the cases, 19 incidents involved counterfeiting (IPR), as in Table 3, including counterfeit brand products and other production origins. Our data showed that well-known brands of roast duck, lamb, and salted chicken had become the main counterfeit objects, improving profit margins. In addition, counterfeit origin refers to the products that counterfeit organic green pork with low price meat or pretend to be the Protected Designation of Origin/Protected Geographical Indication productions.

#### Involving water-injection meat

Water-injected meat is an inferior product obtained by intentionally increasing the water content in the meat to a certain extent to exaggerate the “weight” for illegal profit^59^. As shown in Table 3, 18 cases of water-injected meat were mainly found in beef and pork and usually in the slaughterhouse or during animals’ transport links. We consider such foods to be “diluted” foods of the fraud type. Water-injected meat will not only reduce the edible quality but also easily cause microbial infection and accelerate the deterioration of meat quality. Moreover, water injection to achieve the purpose of using illegal drugs will lead to illegal drug residues in meat, posing a serious threat to human health.

#### Mislabeling/misbranding

Our data showed that 15 cases in Table 3 were related to mislabeling/misbranding. Due to the short shelf life of cooked meat products, behaviors such as changing the label or shelf life were common, especially in promotional products. Such acts may damage consumers’ right to learn the truth, violate the Consumer Protection Law and other regulations, and are illegal. In addition, the label and product information are inconsistent (such as weight fraud), containing undeclared food additives, missing labels, and packaging information, and other events also occur frequently.

#### Certificate fraud

Certificate fraud refers to the event that quarantine officers deliberately issue forgery inspection and quarantine certificates at the veterinary station. This situation may be related to the corrupt behavior of supervisors.

#### Other abnormal food conditions

There were other abnormal food adulterants. Cases recorded substances added to meat products, such as stones, mud, and others. Some would achieve the purpose of increasing weight so that it can be counted as a fraud incident.

### Number of incidents reported by FF for different risk links and locations

Tracking risk links or locations of events may be of great significance to regulatory enforcement and inspections, as well as the identification of vulnerabilities in the food supply chain. For these 1987 FF cases, we classified and counted them in Table 7 based on the sources of FF. The fraudulent behaviors were mainly found in food production (including processing) links (accounting for 15.6%), circulation (including transportation, slaughtering, and marketing) links (72.0%), and catering (catering business) links (12.4%).

**Table 7:** Food risk links or locations involving FF.

Risk links/places involved	Number of cases involved	Percentage (%)
Production links	309	15.6
Medium-sized manufacturing enterprises	122	6.1
Small workshop	175	8.8
Large food company	12	0.6
Circulation links	1431	72.0
Port of entry	773	38.9
Agricultural market	285	14.3
Supermarket chain	98	4.9
In transit or slaughterhouse	97	4.9
Grocery store	55	2.8
Small supermarket	41	2.1
Shopping center	27	1.4
Frozen food store	32	1.6
Online shop	11	0.6
Small street vendor	11	0.6
Supervise the place	1	0.1
Catering links	247	12.4
Small restaurant	135	6.8
Chain restaurant	80	4.0
Deli	26	1.3
Roadside stalls	4	0.2
Online ordering	1	0.1
Canteen	1	0.1
Total	1987	100.0

Source: author’s calculation.

In terms of production link, the main problems found in the most reported small workshops (8.8%) and medium-sized manufacturing enterprises (6.1%) were the forbidden veterinary drugs and misuse of food additives, which may be related to the loose control of raw materials. Due to the hidden environment or location and the lack of legal registration or review for producers, small workshops were occasionally found using industrial substances, heavy use of other animal’s meat or sick livestock and poultry meat for substitution.

In the circulation link, except for customs (38.9%), fraud incidents in domestic meat foods were discovered in agricultural markets (14.3%) and chain supermarkets (4.9%) frequently due to the largest circulation and exchange of commodities. Smuggled meat, unquarantined meat, and water-injected meat were easy to find at other circulation venues, such as in transit or slaughterhouse (4.9%) and frozen food store (1.6%). Grocery stores (2.8%), small supermarkets (2.1%), shopping centers (1.4%), and online stores (0.6%) have detected more fraud cases of unapproved food additives and prohibited veterinary drugs through supervision and inspections.

In terms of catering links, have been discovered and reported: that small restaurants (6.8%), chain restaurants (4.0%), and delis (1.3%) were all found in catering cooked meat products: (1) out-of-range additives and non-edible substances (2) prohibited veterinary drugs in livestock and poultry meat, (3) many cases of substituting beef and mutton with other low-priced animal meats. Frauds, such as expired and unqualified raw materials, existed in the online ordering of meals occasionally.

## Discussion

This study established a meat food fraud risk information database based on 1987 cases of FF through the notification of supervision sampling inspections and custom inspections published by the Chinese authorities, as well as the meat food news reported by the media. Moreover, summarize regional distribution, adulterants, categories involved, categories and sub-categories of foods, risk links, and locations in these events. The results show that food fraud is still a major problem in meat foods. The research has the following policy implications:

First, national supervision and sampling inspection are essential, which can detect and gain a large amount of fraud information. In addition to government information disclosure, media supervision should also be an important means for food safety, especially for food fraud crime supervision and management. The news media in this study recorded (233/250) cases of FF involving “substitution” and (191/869) cases of “artificial enhancement”. Therefore, it is necessary to give full play to the supervision role of the media platform. Adequate media supervision can reduce the cost of consumer complaints and government intervention^60^. The media can also promote the restriction of non-compliant production and solve the problems of market information asymmetry and failure.

Second, some meat products with relatively high prices, such as beef, mutton, brand roast duck products, and the popular sauce-braised meat products, had a high frequency of FF, which is related to the sharp increase in market demand. This shows that we not only need to introduce further, more effective laws to strengthen the key monitoring of these products during the production, circulation, and catering links but also need to strictly implement regulatory measures to increase the cost of crimes. For consumers to learn, fraud identification and risk assessment are also necessary. It is suggested to strengthen the publicity and education on nutrition and safety of diversified meat food, enhance the awareness of green, safe, and balanced nutritious diet, and guide consumers’ preferences and consumption behavior.

Third, we need to strengthen further the risk monitoring of breeding source links and the prevention and control of pollution and strengthen the regulation and raw material control of production enterprises along with business sites (especially small workshops) in the production and circulation links. It is also necessary to guide enterprises themselves to strengthen the control of food fraud risks in key links such as raw material purchase, processing, storage, and transportation. Due to the high proportion of adulteration in circulation, large agricultural markets and supermarkets are encouraged to form stable supply channels by signing agreements with regular breeding bases and conducting supply and marketing cooperation to ensure the safety of meat in the supply chain.

Forth, other suggestions also include continuing to promote the development of low-cost, high-throughput authenticity identification technologies and methods suitable for meat adulteration identification. Strengthen cooperation with meat-importing countries on food safety supervision measures, inspection, quarantine, and certification. Gradually improve the food safety standard system in line with international standards. Learn from relevant countries to implement the traceability “ID code” or traceability system for beef and mutton origin so as to provide consumers with more information and make the product more transparent and credible^61^, etc.

The limitation of this study is that due to inconsistent data attributes, data statistics have not been carried out in the links before entering the market. As the links before and after the entry into the market of Chinese agricultural products are supervised by different departments that is, the agricultural department monitors the risks of livestock products in the breeding and slaughter links, and the market supervision department supervises the products after processing. With the frequent increase of meat supply in the national market, as the data range does not cover the entire industry chain, there may still be food fraud categories and methods that have not been counted.

Moreover, considering the impact of sampling strategies on data results, this study collected all the unqualified information in the domestic supervision and inspection notifications in China as much as possible. However, the use of risk-based food safety supervision and sampling data may still lead to a certain deviation in the fraud research results. When formulating the sampling plan, the authorities extensively solicited the opinions and suggestions from experts, scholars, industries, enterprises, and consumers, taking into account factors such as the regions, covered varieties, enterprises, and safety indicators prescribed by national regulations and prohibited substances comprehensively, so as to carry out standardized sampling inspection. Although these data can basically reflect the situation of food safety, the opinions of stakeholders that give input to the authority’s sampling plan may still affect the balance. For instance, it may lead to the bias of the total sampling amount of each province and then affect the fraud statistics extracted from the data of each province.

## Methods

### Sources of data

As the Chinese market supervision department is mainly responsible for food safety management in the domestic market, the official circulars of supervision and sampling inspection issued by the State Administration for Market Regulation (SAMR) reflect the safety status of most food in the markets and the results of unqualified sampling inspection are of research value^36^. Similarly, the import and export inspection and quarantine department General Administration of Customs of China (GAC) is mainly responsible for inspecting imported food. The official notification of imported food issued by the department is also representative and statistically significant.

Chinese media reports are one of the ways to obtain food fraud news^62^. Due to the limitations of the detection scope, technical means, and cost, it may be insufficient to detect all adulterants via authorities. Then we tracked news articles aggregated by Baidu News (a searchable online database available from https://news.baidu.com/)’ robot programs that contained meat and fraud-related keywords. The results collected by this tool include public network media news released by Sina, Tencent, etc., as well as FoodPartner, ThrowOut, and other professional platforms, and combined all the related events they recorded from 2012 to 2021.

**Fig. 3:**
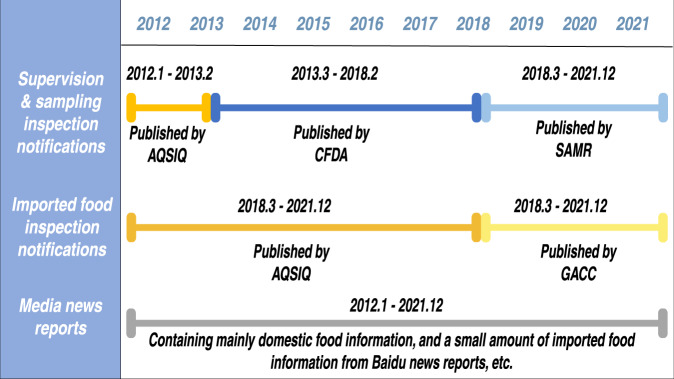
Acquisition sources of statistical data from 2012 to 2021. The timeline columns show the records of official data and news sources during the corresponding time period.

Therefore, we collected data on meat fraud recorded by official circular information and media reports since 2012. According to the data source, with the reform of the relevant food safety supervision departments in China (gradually from multi-part decentralized supervision to unified supervision mode in the past 10 years), the source of official unqualified information comes from the notices issued by different departments respectively: (1) before 2013, the inspection notices of domestic products were generally issued by the Administration of Quality Supervision, Inspection and Quarantine (AQSIQ); (2) and during 2013–2018 issued by the China Food and Drug Administration (CFDA); (3) then published by the SAMR after 2018. 4) The imported food information notification was published by AQSIQ before 2018; 5) and by the General Administration of Customs (GAC) after 2018; 6) The media information was mainly obtained from Baidu search statistics. Figure 1 provides details about the sources of our data. Finally, we created a meat food fraud risk information database based on fraud statistics in China’s meat market. We have obtained 1,987 pieces of FF information on meat products according to the frequency of different substances (that is, the number of adulterations of each substance). Among them, 670(33.7%) cases were obtained from unqualified domestic information of official sampling inspections; 773(38.9%) cases were from imported meat food rejection notifications; 544(27.4%) cases were reported by the media (Table 8 and Fig. 3).

**Table 8:** Sources and characteristics of statistical data.

Source of information	Characteristics	Number of cases involved	Percentage (%)
Domestic food supervision and sampling inspections	It has a strong supervision effect and can play a role in preventing risks from entering the market. Relying on official inspection and testing methods, illegal situations are generally detected in the production, distribution links, and a small number of catering links.	670	33.7
Imported food inspection notifications	Due to the huge size of the Chinese market, the official inspection and quarantine of imported food can also prevent some risks.	773	38.9
Media news reports	Other EMA/FF incidents reported by the media often include a deliberate, intentional substitution of other meat, smuggled meat and by-products not entered through customs, and incidents escaping official supervision and sampling inspections. Most of them are discovered before or after consumption.	544	27.4
Total	/	1987	100.0

Source: author’s calculation.

### Classification of data

Based on the three data information sources described above, we filtered and classified the tracked data (done by the first, third, and fifth authors). To begin with, we accumulated information about domestic food supervision and sampling inspection notifications (including food categories, origins, inspection results, unqualified ingredients, etc.) through the public data, in which we directly sorted out our data related to meat food fraud.

Moreover, those imported meat foods were sorted from the existing information from the imported food inspection notifications (including food categories, origin countries, and reasons for entry disqualification) by screening relevant FF data.

As for media news reports, in our collation of news events, we mainly searched for possible keywords related to “meat” or “fraud” through the engine. However, that was not enough. Mainly based on the label classification automatically marked by news search engines, such as livestock and poultry meat, adulteration, counterfeit, food crime, and illegal addition, we combined all the suggested keywords to collect data. We manually checked the time, place, source, and other factors of all retrieved events to ensure that these events will not be counted repeatedly. Additionally, we verified the authenticity of the data and filtered out fake news and rumor information that have been refuted in this period.

Finally, we unified the classification method and format for data (done by the first, third, and fourth authors). The classification mainly relied on the China Food Production License Catalogue^63^ also concerning the Food fraud technical document issued by GFSI^64^ and academic literature^1,32^^,33^. Types of meat food fraud were divided into the following: mislabeling/misbranding, counterfeiting (IPR), substitution, artificial enhancement, dilution, illegal imports, certificate fraud, and others. The descriptions of fraud types and classifications are shown in Table 9.

**Table 9:** Description of food fraud types in meat foods.

Fraud types	Descriptions	Example
Mislabeling/misbranding	Including labels inconsistent with product information, undeclared food additives, and missing labels	Missing packaging information; changing the packaging label or scan the barcode of the expired meat to the market; unlabeled bulk meat products; selling cooked food without removing the weight of the box
Artificial enhancement	Adding unapproved food additives, non-edible substances, prohibited veterinary drugs, and industrial substances to artificially enhance product quality or other attributes.	Prohibited veterinary drug metronidazole was detected in chicken meat sold at agricultural markets; adding food additives carmine pigment, nitrite, or non-edible substances Auramine O to the braised meat products; pork and trotter processing using industrial rosin and hydrogen peroxide
Counterfeiting (IPR)	Unauthorized parties fraudulently label products as brands or imitate other origins.	Counterfeiting brand roast duck with ordinary products; counterfeiting meat products from other origins
Substitution	Substituting the original animal components of food with spoiled, untested, unclean animal meat or other animal-derived or plant-derived components.	Substituting ordinary or better pork and processed meat preparations with expired, smuggled, dead livestock and poultry meat; substituting pork and duck for more expensive beef and mutton; using corn starch instead of beef to process meat products
Dilution	Increasing the overall weight or volume of the product by adding water to the meat.	Injecting water in cattle to increase beef weight during transit link or at slaughterhouses
Illegal imports	Imported food has no official inspection and quarantine certificate, approval certificate, or origin certificate, has not obtained inspection and quarantine access, or the goods do not conform to the certificate.	Pork, trotter, and pig kidney without official quarantine and origin certificate; smuggled beef and chicken feet
Certificate fraud	Forging the inspection and quarantine certificates of meat products.	Forging pig quarantine certificate
Other	Foreign objects and other conditions	Presence of other foreign objects that affect weight, such as sand

### Reporting summary

Further information on research design is available in the Nature Research Reporting Summary linked to this article.

## Supplementary information

Reporting Summary

Supplementary Material

## Data Availability

The original data used in this article are all from public websites. The data set generated and analyzed during the current research period can be obtained from the corresponding author upon request.
