# Supplementary material for: Meat food fraud risk in Chinese markets 2012–2021
Source: NPJ Sci Food. 2023 Apr 3;7:12. doi: 10.1038/s41538-023-00189-z (PMC10070328; doi:10.1038/s41538-023-00189-z)
Supplement: Supplementary file 2 — Supplementary Material [file 41538_2023_189_MOESM2_ESM.pdf]

Supplementary Table 1: Specific FF drugs involving prohibited drugs in meat foods

Veterinary/pesticides drugs involved	Beef	Chicken	Pork	Other livestock by-products	Mutton	Sauce-braised meat products	Pork liver	Other poultry by-products	Sauce-braised meat products (homemade)	Cured meat products	Pig kidney	Duck	Sheep kidney	Chicken liver	Smoked barbecue meat products	Other poultry	Quick-frozen prepared meat products	Other cooked meat (homemade)	Beef liver	Smoked boiled sausage and ham products	Number of cases involved	Percentage (%)
Doping substances	72		19	29	29	18	3		13		9		5		1			1	1		200	47.4
Clenbuterol	67		1	1	29	1	1		6		1		5					1	1		114	27.0
Ractopamine	5		13(10 from imported food)	26(22 from imported food)		17			4		8(6 from imported food)				1						74	17.5
Salbutamol			5	2			2		3												12	2.8
Aantibiotic	10	41	34	7	5	10	11	10	3	15		4		3	4	4	2			1	164	38.9
Chloramphenicol	4(3 from imported food)	14	8	1		10		3	3	15		2			4	2	2			1	69	16.4
Ofloxacin	4	11	14	1	2		4	1													37	8.8
Furazolidone Metabolites	2	7	7		1		4	6				2		3		2					34	8.1
Nitrofurazone metabolites		1	4	4				2(1 from imported food)													11	2.6
Norfloxacin		3		1	2																6	1.4
Metronidazole		2																			2	0.5
Pefloxacin		1	1																		2	0.5
3-Amino-2-oxazolidinone		1																			1	0.2
Furatadone metabolites		1																			1	0.2

Nitrofurantoin metabolites							1														1	0.2
Banned pesticides	2	15	4	2	2	1	9	6				5		1							47	11.1
Sodium pentachlorophenate	2	15	4	2	2	1	9	6				5		1							47	11.1
Antiviral Agents		7	1		1									1							10	2.4
Amantadine		7												1							8	1.9
Ribavirin			1		1																2	0.5
Psychotropic drugs			1																		1	0.2
Chlorpromazine			1																		1	0.2
Total	84	63	59	38	37	29	23	16	16	15	9	9	5	5	5	4	2	1	1	1	422	100.0

Source: author's calculation.

Supplementary Table 2: Specific FF substances involved in non-compliant use of food additives in meat foods

Food additives involved	Sauce-braised meat products	Sauce-braised meat products (homemade)	Beef	Cured meat products	Dried meat products	Mutton	Other cooked meat (homemade)	Smoked boiled sausage and ham products	Other livestock by-products	Meat enema (homemade)	Smoked barbecue meat products	Pork	Pork liver	Number of cases involved	Percentage (%)
Colorants	62	14	2	14	3	2		6			3	1		107	36.0
Carmine	34	7	1	12	1	2		6			2			65	21.9
sunset yellow	15	6			1						1	1		24	8.1
lemon yellow	8	1			1									10	3.4
caramel coloring	2													2	0.7
Allura Red	2													2	0.7
Monascus red	1													1	0.3
caramel colour				1										1	0.3
Pigment Red 2G				1										1	0.3
Colorant (unknown)			1											1	0.3
Color protectant		46	4			3	7		2	3			1	66	22.2
nitrite		46	3			3	7		2	3			1	65	21.9
casing salt			1											1	0.3
Preservatives	17	7	2	19	14	2				1	1			63	21.2
benzoic acid	17	6	2	5	14	2				1	1			48	16.2
Sorbic acid and its potassium salts				13										13	4.4
sorbic acid		1		1										2	0.7
Bleaching agents			48									1		49	16.5
sulfur dioxide			48									1		49	16.5
Sweeteners	5							1						6	2.0
Sodium Saccharin	2							1						3	1.0
Acesulfame potassium	2													2	0.7
Cyclamate	1													1	0.3
Swelling agent			1						3					4	1.4
sodium bicarbonate			1						1					2	0.7
Aluminium Ammonium Sulfate									2					2	0.7
Moistrue retention agents			1			1								2	0.7
Sodium Phosphate			1			1								2	0.7

Total	84	67	58	33	17	8	7	7	5	4	4	2	1	297	100.0

Source: author's calculation.

Supplementary Table 3: Specific FF substances involved in industrial substances and other non-edible substances

Non-edible substances involved	Other livestock by-products	Other poultry by-products	Sauce-braised meat products	Other cooked meat (homemade)	Pork	Sauce-braised meat products (homemade)	Beef	Cured meat products	Duck	Mutton	Other meat	Other poultry	Meat enema (homemade)	Aspic, skin jelly (homemade)	Pork liver	Smoked boiled sausage and ham products	Quick-frozen prepared meat products	Number of cases involved	Percentage (%)
Industrial substances	32	21	9	1	11	3	2	5	4		1	1	1	1		1		93	62.0
Hydrogen peroxide	12	11	1		1													25	16.7
industrial rosin	7		1		7	2			4			1						22	14.7
Industrial sodium nitrite	2		4		2		1	5			1					1		16	10.7
formaldehyde	7	7		1									1					16	10.7
Industrial sodium hydroxide	4	1	1															6	4.0
industrial gelatin			1		1		1							1				4	2.7
Industrial Ferrous Sulfate		1																1	0.7
Industrial Magnesium Chloride		1																1	0.7
industrial dyes			1															1	0.7
Industrial Pigment						1												1	0.7
Other non-edible substances	3	2	10	11		7	6	3	3	6	2	2			1		1	57	38.0
poppy shell	3	2	6	11		7	5		3	6	2	2						47	31.3

Acid Orange II			1					2										3	2.0
Alkaline Bright Yellow O			2															2	1.3
Borax							1										1	2	1.3
Dichlorvos								1										1	0.7
Congo red															1			1	0.7
Sudan red			1															1	0.7
Total	35	23	19	12	11	10	8	8	7	6	3	3	1	1	1	1	1	150	100.0

Supplementary Table 4: Specific FF substances involving other low-priced alternative meat and ingredients

Low-priced substitutes involved	Pork	Sauce-braised meat products	Beef	Cured meat products	Meat from other livestock	Chicken	Quick-frozen prepared meat products	Mutton	Dried meat products	Smoked barbecue meat products	Other livestock by-products	Other cooked meat (homemade)	Other poultry by-products	Smoked boiled sausage and ham products	Number of cases involved	Percentage (%)
Sick / dead livestock or poultry meat	28	3		6	1	1		2	1		1				43	43.4
sick / pig	28	3		6				1			1				39	39.4
Sick / dead livestock (except pig)					1			1	1						3	3.03
Sick / dead poultry						1									1	1.01
Expired meat / spoiled meat	11	3	5			2	4			3		1	1	1	31	31.3
Expired meat	8	3	5			2	4			3			1	1	27	27.3
spoiled meat	3											1			4	4.0
Entrained low-quality meat		3				2		1				1			7	7.1
meat from lymph or other organs		3						1				1			5	5.05
chicken skin						1(1 from imported food)									1	1.0
Chicken breast or feet						1(1 from imported food)									1	1.01
Meat of unknown origin	2		2			1		1							6	6.1
Meat of unknown origin			1			1		1							3	3.03
Unquarantine meat	2		1												3	3.0
Poisonous meat ingredients					5										5	5.1
Poisonous meat contaminated with succinylcholine or cyanide					5										5	5.05

Plant-derived ingredients		1							2		1				4	4.0
soy flour									2						2	2.0
unknown											1				1	1.0
corn starch		1													1	1.0
Unclean meat		1		1			1								3	3.0
Unclean meat		1		1			1								3	3.0
Total	41	11	7	7	6	6	5	4	3	3	2	2	1	1	99	100.0

Source: author's calculation.
